# Impact of pharmacist active consultation on clinical outcomes and quality of medical care in drug-induced liver injury inpatients in general hospital wards: A retrospective cohort study

**DOI:** 10.3389/fphar.2022.972800

**Published:** 2022-08-30

**Authors:** Dongxuan Li, Jie Dong, Xin Xi, Guili Huang, Wenjun Li, Cheng Chen, Jun Liu, Qian Du, Songqing Liu

**Affiliations:** ^1^ Department of Pharmacy, The Third Affiliated Hospital of Chongqing Medical University, Chongqing, China; ^2^ College of Pharmacy, Chongqing Medical University, Chongqing, China; ^3^ Center for Medical Information and Statistics, The Third Affiliated Hospital of Chongqing Medical University, Chongqing, China

**Keywords:** drug-induced liver injury, clinical pharmacist, pharmacist active consultation, Roussel Uclaf Causality Assessment Method (RUCAM), patient recovery, medical care quality

## Abstract

The utility of pharmacist consultation for drug-induced liver injury (DILI) management has not been explored. This retrospective cohort study evaluated the impact of a pharmacist active consultation (PAC) service on the management and outcome in patients with DILI. Consecutive patients meeting clinical biochemical criteria for DILI were enrolled at a tertiary teaching hospital between 1 January 2020 and 30 April 2022. The Roussel Uclaf Causality Assessment Method was used to assess causality between drug use and liver injury for each suspected DILI patient. Included patients were grouped according to whether they received PAC, and a proportional hazard model with multivariate risk adjustment, inverse probability of treatment weighting (IPTW), and propensity score matching (PSM) was used to assess DILI recovery. In the PSM cohort, the quality of medical care was compared between PAC and no PAC groups. A total of 224 patients with DILI (108 who received PAC and 116 who did not) were included in the analysis. Of these patients, 11 (10%) were classified as highly probable, 58 (54%) as probable, and 39 (36%) as possible DILI in the PAC group, while six patients (5%) were classified as highly probable, 53 (46%) as probable, and 57 (49%) as possible DILI in the no PAC group (*p* = 0.089). During patient recovery, PAC was associated with a ∼10% increase in the cumulative 180-day recovery rate. The PAC group had a crude hazard ratio (HR) of 1.73 [95% confidence interval (CI): 1.23–2.43, *p* = 0.001] for DILI 180-day recovery, which remained stable after multivariate risk adjustment (HR = 1.74, 95% CI: 1.21–2.49, *p* = 0.003), IPTW (HR = 1.72, 95% CI: 1.19–2.47, *p* = 0.003), and PSM (HR = 1.49, 95% CI: 1.01–2.23, *p* = 0.046). In the PSM cohort, PAC was more likely to identify suspect drugs (90% vs. 60%, *p* < 0.001) and lead to timely withdrawal of the medication (89% vs. 57%, *p* < 0.001). Thus, PAC is associated with a better quality of medical care for patients with DILI and can improve patient outcomes.

## Introduction

Drug-induced liver injury (DILI) is an adverse reaction induced by small-molecule drugs, biological agents, traditional Chinese medicines, and herbal and dietary supplements (Yu et al., 2017). The incidence of DILI ranges from 12.0/1,00,000 to 19.1/1,00,000 in the general population and varies according to region, study design, and patient inclusion and exclusion criteria (Sgro et al., 2002; Suk et al., 2012; Bjornsson et al., 2013). DILI is an increasingly important clinical problem for which diagnosis and treatment guidelines have been developed in recent years (Yu et al., 2017; European Association for the Study of the Liver. Electronic address et al., 2019; Chalasani et al., 2021; Devarbhavi et al., 2021). However, there are more than 1,000 drugs and dozens of diseases that can cause liver damage (Giannini et al., 2005; Malakouti et al., 2017; Thakkar et al., 2020), and the diagnosis of DILI mainly relies on the exclusion of other etiologies of liver disease and identification of suspect drugs, which requires clinical and pharmaceutical expertise. As such, the management of DILI patients remains challenging, especially for inexperienced medical personnel.

Clinical pharmacists are an important part of the patient-centered diagnosis and treatment team with professional pharmacy knowledge and the ability to provide comprehensive medication management (Saseen et al., 2017). Pharmacists have played a positive role in the prevention of cardiovascular events; anticoagulant treatment; preconception care; and management of infection, pain, cancer treatment adverse reactions, and type 2 diabetes (Saokaew et al., 2010; Dunn et al., 2015; DiPietro Mager, 2016; Sakeena et al., 2018; Durrer et al., 2021; Homan et al., 2021; Thapa et al., 2021). However, there have been no studies to date evaluating the impact of pharmacist involvement in the management of patients with DILI.

In order to explore and optimize the model of DILI management, we established a pharmacist active consultation (PAC) service at our hospital that consists of spontaneous active consultation conducted by clinical pharmacists for suspected DILI patients, with the intent of providing optimal and timely treatment recommendations. Herein, we describe the impact of PAC on DILI patient outcomes.

## Methods

This study was conducted at the Third Affiliated Hospital of Chongqing Medical University, a 1350-bed tertiary teaching hospital in Chongqing, China, with approximately 40,000 annual patient admissions.

### PAC service for DILI patients

On 1 March 2021, clinical pharmacists at our center began implementing the PAC service for hospitalized patients with suspected DILI. Clinical pharmacists identified patients with DILI according to the following clinical biochemistry criteria: 1) alanine aminotransferase (ALT) ≥5× the upper limit of the normal range (ULN), 2) ALT ≥3× ULN and total bilirubin >2 × ULN, or 3) alkaline phosphatase (ALP) ≥2 × ULN and gamma-glutamyl transferase >1 × ULN (Aithal et al., 2011).

Every working day, a clinical pharmacist reviewed each case that met the abovementioned criteria along with medical history, medication history, and LiverTox (https://www.ncbi.nlm.nih.gov/books/NBK547852) and immediately initiated PAC for patients with suspected DILI. This included the following steps: 1) explaining the possible reason for liver injury to patients and doctors; 2) identifying the possible causative drugs; 3) discontinuing, adjusting the dose of, or continuing treatment with the drug depending on the patient’s condition; 4) selecting appropriate drugs for liver injury treatment; 5) conducting a 10-min education session for the patient; and 6) monitoring changes in liver function parameters and proposing interventions when necessary. Clinical pharmacists participated in routine ward rounds.

### Study design and patient population

Using a retrospective cohort study design, consecutive patients were enrolled from 1 January 2020 to 30 April 2022 if they had at least one liver function test meeting one of the aforementioned clinical biochemistry criteria for DILI.

Patients with unambiguous alternative etiologies for liver injury were excluded; these included liver injury in infants, viral liver disease, alcoholic liver disease, autoimmune liver disease, cholestatic liver diseases, infection (e.g., liver abscess, sepsis), hemodynamic abnormality, hepatobiliary pancreatic tumor, pancreatitis, direct liver injury, osteopathy, liver cirrhosis, intestinal disease, and other nondrug or unknown causes of liver injury (Giannini et al., 2005; Malakouti et al., 2017; European Association for the Study of the Liver. Electronic address et al., 2019; Chalasani et al., 2021; Devarbhavi et al., 2021). Patients admitted to the hospital’s Hepatology Department were excluded as they were treated by a specialist experienced in DILI management, and, therefore, PAC was not performed by the clinical pharmacist. Additionally, as the Chinese Society of Hepatology strongly recommends the use of the Roussel Uclaf Causality Assessment Method (RUCAM) to establish causality in the clinical diagnosis of DILI, this was applied to each suspected case (Danan and Teschke, 2016; Yu et al., 2017). Patients were classified as highly probable (RUCAM score ≥ 9), probable (6–8), possible (3–5), unlikely (1 or 2), or excluded (≤0). Patients who were categorized as “unlikely” and “excluded” (<3) and those without follow-up liver function test data were excluded from the analysis. The remaining patients were divided into no PAC and PAC groups based on whether they received the PAC intervention.

### Ethics

This study was approved by the Third Affiliated Hospital of Chongqing Medical University Review Board with a waiver for informed consent (No. 2021-37).

### Definitions

The R-value [(ALT/ALT ULN)/(ALP/ALP ULN)] was used to categorize the injury pattern of DILI as hepatocellular (R ≥ 5), cholestatic (R ≤ 2), or mixed (2 < R < 5) (Danan and Benichou, 1993; Aithal et al., 2011; Danan and Teschke, 2016). The severity of DILI was categorized into four grades, namely, mild, moderate, severe, and fatal/transplantation, according to the DILI severity grading scale developed by the International DILI Expert Working Group (Supplementary Table S1) (Aithal et al., 2011).

### Patient recovery and follow-up

Patient recovery was defined as a return to normal of the patient’s serum biochemical parameters (1 × ULN) (Ashby et al., 2021). As patients with DILI whose liver function did not return to normal for >6 months were considered to have a chronic liver injury (Yu et al., 2017), we set 180 days as the cutoff point for follow-up. Time to recovery or follow-up time was calculated in days from the day the patient met the clinical biochemical criteria for DILI to the date of normalization of liver serum biochemical parameters (1 × ULN) or the last day of follow-up. Patients with serum ALT, aspartate aminotransferase, ALP, or bilirubin that did not return to 1 × ULN were censored at the date of their last recorded follow-up.

### Inpatient DILI management quality

Seeking expert consultation is helpful to ascertain the diagnosis of DILI and attribute causality to a suspect drug (Chalasani et al., 2021). In this study, the expert was a pharmacologist or hepatologist. The appearance of the term “drug-induced liver injury” in medical records indicated that the physician was aware of the possibility of DILI, and the appearance of a specific drug name indicated that the causative drug had been identified.

Timely discontinuation was defined as discontinuation of the suspect drug within 24 h of the patient meeting the clinical biochemical criteria for DILI. Drugs, treatment measures, and liver function monitoring intervals were recorded for each group to assess differences in patient management. The time interval from meeting the clinical biochemical criteria for DILI to receiving expert consultation was calculated in hours for each patient to evaluate the efficiency of PAC service delivery.

### Outcome assessment

The primary outcome of this study was a 180-day patient recovery rate and hazard ratio (HR). The secondary outcome was the quality of inpatient DILI management.

### Data collection

Data were obtained from patients’ electronic and paper medical records and entered into a standardized case report form, which included demographics, comorbidities, suspect drug, DILI clinical characteristics, treatment and management measures, and clinical outcomes.

### Statistical analysis

Continuous variables were compared with the Student’s *t*-test when normally distributed or with the Mann–Whitney U test. The chi-squared test or Fisher’s exact test was used for categorical variables where appropriate. Kaplan–Meier survival analysis with the log-rank test was performed and cumulative events in the 180-day follow-up period were compared between the groups.

In the Cox proportional hazards model, potential predictors of 180-day recovery from liver injury were first assessed in a univariate analysis. Covariates were included in the final model if the *p*-value was ≤0.2 or if they were clinically important. Cox regression analysis was performed to assess the impact of PAC on the rate of 180-day recovery from liver injury, with results presented as HR with a 95% confidence interval (CI).

In a second analysis, using the variables from the univariate analysis, the inverse probability of treatment weighting (IPTW) and propensity score matching (PSM) were performed to control for selection bias and potential confounding factors between groups. A propensity score (PS) was calculated for each patient as the predicted probability of PAC from multivariate logistic regression. Based on individual PSs, a Cox regression model was generated using the IPTW approach with PAC as the only covariate. In addition, based on individual PSs, we performed a 1:1 nearest neighbor matching without replacement with a caliper width of 0.2, yielding a PSM cohort. A Cox regression model was generated for the matched cohort with PAC as the only covariate. Standardized mean differences were used to assess the performance of the IPTW and PSM, with a value <0.10 considered as evidence of balance (Austin and Stuart, 2015). Finally, using PSM cohorts, differences in management quality for patients with DILI were assessed to determine the utility of PAC services.

All statistical tests were two-sided, and *p* < 0.05 was set as the level of statistical significance. Data were analyzed using R v4.1.0 (R Foundation for Statistical Computing, Vienna, Austria).

## Results

### Study population and clinical characteristics

**FIGURE 1:**
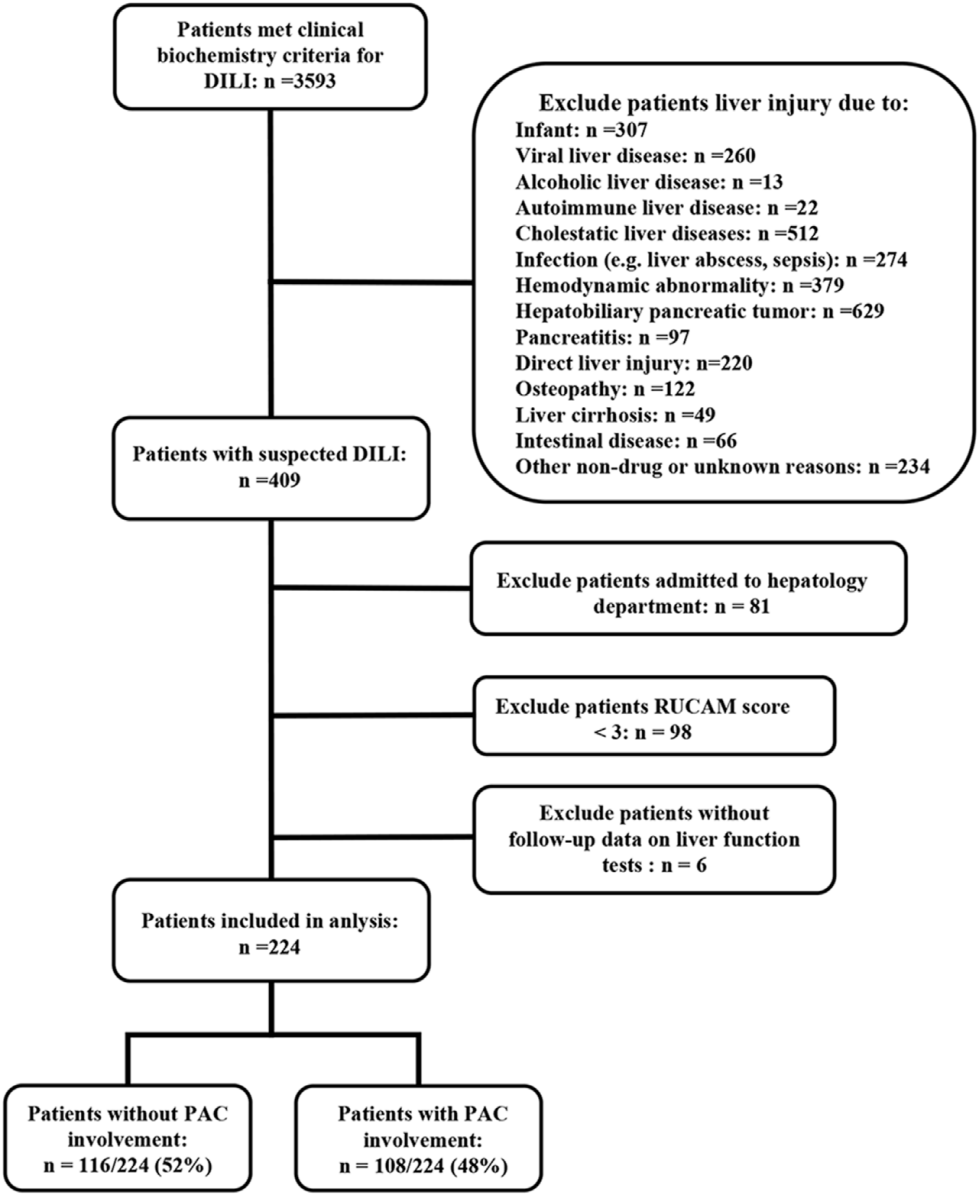
Flow diagram of patients included in the study. Abbreviations: DILI, drug-induced liver injury and PAC, pharmacist active consultation.

Of the 3,593 patients meeting the clinical biochemical criteria for DILI, 3,184 were excluded. Of the remaining 224 eligible patients, 116 (52%) were assigned to the no PAC group and 108 (48%) to the PAC group (Figure 1). Using the updated RUCAM causality assessment method, 11 patients (10%) were classified as highly probable, 58 (54%) as probable, and 39 (36%) as possible DILI in the PAC group and six patients (5%) were classified as highly probable, 53 (46%) as probable, and 57 (49%) as possible DILI in the no PAC group (*p* = 0.089). Among patients with possible alternative causes of liver injury, the diagnosis was mostly viral hepatitis and recent hemodynamic abnormality (Supplementary Table S2).

Patient baseline demographic and clinical characteristics are shown in Table 1. There were significant differences between groups in the DILI onset site (*p* = 0.023), jaundice (*p* = 0.001), anorexia (*p* = 0.008), nausea (*p* = 0.010), vomiting (*p* = 0.036), and severity grade (*p* < 0.001). Among the 224 patients included in the analysis, because of the use of multidrug combinations, 260 drugs were considered causative drugs for DILI; the most common drug classes were “antineoplastic and immunomodulating agents” (Supplementary Table S3).

**TABLE 1:** Patient baseline and drug-induced liver injury clinical characteristics.

Characteristic	All patients (*N* = 224)	No PAC service (*N* = 116)	PAC service (*N* = 108)	*p*-value
Age, median, years [IQR]	55.0 [46.0, 65.0]	55.5 [45.0, 65.0]	55.0 [49.8, 64.0]	0.921
Age ≥ 60 years	84 (37.5)	50 (43.1)	34 (31.5)	0.075
Male	130 (58.0)	68 (58.6)	62 (57.4)	0.893
DILI onset site				0.023**
Community-acquired^a^	101 (45.1)	61 (52.6)	40 (37.0)	
Hospital-acquired	123 (54.9)	55 (47.4)	68 (63.0)	
Drinking history	25 (11.2)	17 (14.7)	8 (7.4)	0.094
Comorbidity				
Cardiovascular diseases	63 (28.1)	35 (30.2)	28 (25.9)	0.552
Nervous system disease	34 (15.2)	18 (15.5)	16 (14.8)	>0.999
Chronic lung disease	18 (8.0)	8 (6.9)	10 (9.3)	0.625
Chronic kidney disease	3 (1.3)	3 (2.6)	0 (0.0)	0.248
Liver underlying disease^b^	28 (12.5)	16 (13.8)	12 (11.1)	0.687
Gastrointestinal diseases	29 (12.9)	16 (13.8)	13 (12.0)	0.843
Autoimmune disease	14 (6.2)	7 (6.0)	7 (6.5)	>0.999
Diabetes	13 (5.8)	7 (6.0)	6 (5.6)	>0.999
Hyperlipidemia	12 (5.4)	6 (5.2)	6 (5.6)	>0.999
Traumatic diseases	17 (7.6)	10 (8.6)	7 (6.5)	0.619
Malignant tumor	80 (35.7)	40 (34.5)	40 (37.0)	0.780
Biochemical patterns of DILI				0.352
Hepatocellular type	130 (58.0)	64 (55.2)	66 (61.1)	
Mixed type	43 (19.2)	21 (18.1)	22 (20.4)	
Cholestatic type	51 (22.8)	31 (26.7)	20 (18.5)	
Accompanying symptoms				
Jaundice	30 (13.4)	24 (20.7)	6 (5.6)	0.001**
Anorexia	23 (10.3)	18 (15.5)	5 (4.6)	0.008**
Nausea	17 (7.6)	14 (12.1)	3 (2.8)	0.010**
Vomiting	9 (4.0)	8 (6.9)	1 (0.9)	0.036*
Abdominal discomfort	16 (7.1)	11 (9.5)	5 (4.6)	0.198
Rash	8 (3.6)	2 (1.7)	6 (5.6)	0.159
Severity grading				<0.001***
Mild	160 (71.4)	71 (61.2)	89 (82.4)	
Moderate	57 (25.4)	38 (32.8)	19 (17.6)	
Severe	7 (3.1)	7 (6.0)	0 (0.0)	

Data are presented as no. of patients (%) unless otherwise specified.

a Community-acquired DILI was defined as a liver injury occurring in a community setting with the patient admitted to the hospital on the first liver biochemical test above the threshold.

b Liver malignancies were not included in underlying liver disease but were classified as malignant tumors.

Abbreviations: DILI, drug-induced liver injury; IQR, interquartile range; and PAC, pharmacist active consultation.

### Pharmacist interventions in the PAC group

For the 108 patients in the PAC group, a clinical pharmacist made treatment recommendations based on the patients’ condition (Table 2). Clinical pharmacists conducted 10-min patient education sessions for 89 patients (82%); the remaining 19 (18%) were unable to communicate because of the loss of consciousness. Clinical pharmacists and physicians discussed the management of DILI for all patients in the PAC group in order to collaboratively develop an optimal regimen.

**TABLE 2:** Recommendations of clinical pharmacists on the management of drug-induced liver injury.

Recommendation	No. (%) of 108 PAC cases
Without intervention—patient education only^a^	11 (10.2)
Discontinue suspect drug	56 (51.9)
Adjust drug dose	2 (1.9)
Switch to alternative medicines	6 (5.6)
Cautious drug rechallenge	10 (9.3)
Add hepatoprotective drugs	68 (63.0)
Treatment with glucocorticoids^b^	7 (6.5)
Screening for viral hepatitis	15 (13.9)
Screening for autoimmune liver disease	10 (9.3)
Abdominal imaging	9 (8.3)
Repeat liver biochemistry in 2–4 days	97 (89.8)

a The reason for no intervention was that the clinical pharmacist believed that the management of drug-induced liver injury was appropriate and no further intervention was required.

b Glucocorticoids were used to treat immune checkpoint inhibitor-related hepatotoxicity.

Abbreviation: PAC, pharmacist active consultation.

### IPTW weighting and PSM cohort

**FIGURE 2:**
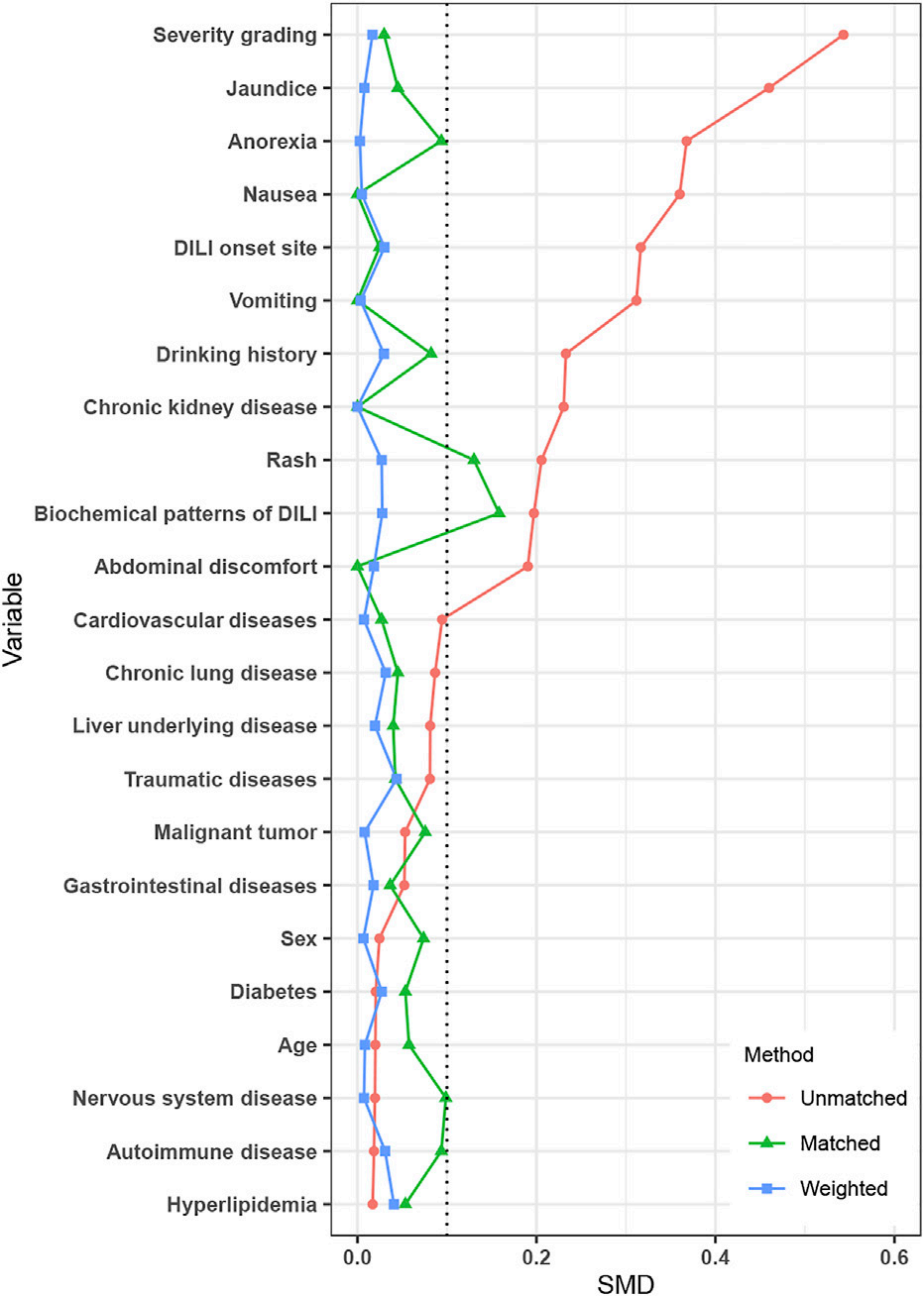
Standardized mean difference between the no PAC and PAC groups in unmatched, PSM, and IPTW cohorts. Abbreviations: PAC, pharmacist active consultation; PSM, propensity score matching; and IPTW, inverse probability of treatment weighting.

After IPTW, covariates were well-balanced between the PAC and no PAC groups (Figure 2). A total of 164 patients were matched by PSM (82 per group), which improved the balance of covariates between groups (Table 3).

**TABLE 3:** Patient baseline and drug-induced liver injury characteristics in the propensity score-matched cohort.

Characteristic	All patients (*N* = 164)	No PAC service (*N* = 82)	PAC service (*N* = 82)	*p*-value
Age, median, years [IQR]	55.0 [45.8, 64.0]	55.0 [43.5, 65.0]	55.0 [49.2, 63.8]	0.760
Age ≥ 60 years	58 (35.4)	34 (41.5)	24 (29.3)	0.141
Male	92 (56.1)	47 (57.3)	45 (54.9)	0.875
DILI onset site				>0.999
Community-acquired^a^	71 (43.3)	35 (42.7)	36 (43.9)	
Hospital-acquired	93 (56.7)	47 (57.3)	46 (56.1)	
Drinking history	16 (9.8)	9 (11.0)	7 (8.5)	0.793
Comorbidity				
Cardiovascular diseases	47 (28.7)	23 (28.0)	24 (29.3)	>0.999
Nervous system disease	27 (16.5)	12 (14.6)	15 (18.3)	0.674
Chronic lung disease	13 (7.9)	7 (8.5)	6 (7.3)	>0.999
Liver underlying disease^b^	17 (10.4)	9 (11.0)	8 (9.8)	>0.999
Gastrointestinal diseases	21 (12.8)	10 (12.2)	11 (13.4)	>0.999
Autoimmune disease	12 (7.3)	7 (8.5)	5 (6.1)	0.766
Diabetes	9 (5.5)	4 (4.9)	5 (6.1)	>0.999
Hyperlipidemia	9 (5.5)	4 (4.9)	5 (6.1)	>0.999
Traumatic diseases	15 (9.1)	8 (9.8)	7 (8.5)	>0.999
Malignant tumor	61 (37.2)	29 (35.4)	32 (39.0)	0.747
Biochemical patterns of DILI				0.543
Hepatocellular type	43 (26.2)	23 (28.0)	20 (24.4)	
Mixed type	91 (55.5)	42 (51.2)	49 (59.8)	
Cholestatic type	30 (18.3)	17 (20.7)	13 (15.9)	
Accompanying symptoms				
Jaundice	12 (7.3)	6 (7.3)	6 (7.3)	>0.999
Anorexia	12 (7.3)	7 (8.5)	5 (6.1)	0.766
Nausea	6 (3.7)	3 (3.7)	3 (3.7)	>0.999
Vomiting	2 (1.2)	1 (1.2)	1 (1.2)	>0.999
Abdominal discomfort	11 (6.7)	6 (7.3)	5 (6.1)	>0.999
Rash	6 (3.7)	2 (2.4)	4 (4.9)	0.682
Severity grading				>0.999
Mild	160 (71.4)	71 (61.2)	89 (82.4)	
Moderate	35 (21.3)	18 (22.0)	17 (20.7)	

Data are presented as no. of patients (%) unless otherwise specified.

a Community-acquired DILI was defined as a liver injury occurring in a community setting with the patient admitted to the hospital on the first liver biochemical test above the threshold.

b Liver malignancies were not included in underlying liver disease but were classified as malignant tumors.

Abbreviations: DILI, drug-induced liver injury; IQR, interquartile range; and PAC, pharmacist active consultation.

### Outcomes

**FIGURE 3:**
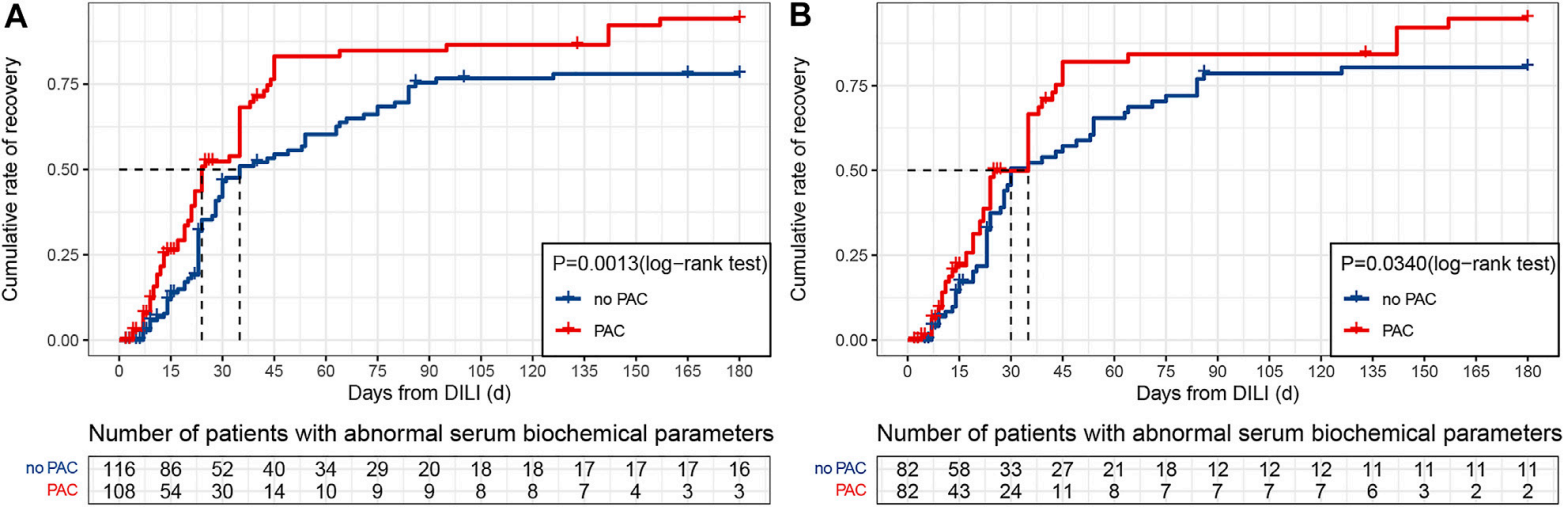
Kaplan–Meier cumulative event rates for time to recovery. **(A)** Original cohort. **(B)** PSM cohort. Abbreviations: DILI, drug-induced liver injury and PAC, pharmacist active consultation.

The cumulative recovery rate over the 180 days follow-up period was 96.3% in the PAC group and 86.2% in the no PAC group (Figure 3A). That is, PAC increased the recovery rate by approximately 10%; this increase persisted after controlling for confounding factors (Figure 3B). We also examined 19 patients in the original cohort whose liver function did not return to normal within 180 days; information on these patients is shown in Supplementary Table S4.

Ten covariates were included in the multivariate analysis with a Cox proportional hazards model that included PAC, age ≥ 60 years, nervous system disease, chronic kidney disease, underlying liver disease, autoimmune disease, biochemical patterns of DILI, nausea, abdominal discomfort, and severity grade. PAC was associated with a higher crude HR (1.73, 95% CI: 1.23–2.43, *p* = 0.001) and adjusted HR (1.74, 95% CI: 1.21–2.49, *p* = 0.003) for DILI recovery (Table 4), whereas no statistically significant differences between the PAC and no PAC groups were observed for the other nine covariates. The higher HRs for the PAC group persisted with IPTW (1.72, 95% CI: 1.19–2.47, *p* = 0.003) and PSM (1.49, 95% CI: 1.01–2.23, *p* = 0.046).

**TABLE 4:** Univariate and multivariate analyses to predict recovery in drug-induced liver injury patients.

Variable	Univariate analysis		Multivariate analysis	
	Crude HR (95% CI)	*p*-value	aHR (95% CI)	*p*-value
PAC	1.73 (1.23–2.43)	0.001	1.74 (1.21–2.49)	0.003**
Age ≥ 60 years	0.78 (0.55–1.11)	0.166	0.90 (0.62–1.31)	0.595
Female	0.86 (0.61–1.20)	0.360	—	—
DILI onset site^a^				
Hospital-acquired	0.90 (0.65–1.26)	0.552	—	—
Community-acquired	Reference			
Drinking history	0.80 (0.47–1.36)	0.409	—	—
Comorbidity				
Cardiovascular diseases	0.99 (0.68–1.42)	0.939	—	—
Nervous system disease	0.67 (0.42–1.08)	0.097	0.71 (0.43–1.16)	0.172
Chronic lung disease	0.84 (0.41–1.73)	0.642	—	—
Chronic kidney disease	0.23 (0.03–1.67)	0.147	0.30 (0.04–2.31)	0.249
Liver underlying disease^b^	1.42 (0.89–2.27)	0.140	1.45 (0.88–2.38)	0.146
Gastrointestinal diseases	1.07 (0.67–1.72)	0.778	—	—
Autoimmune disease	1.63 (0.88–3.04)	0.123	1.52 (0.80–2.86)	0.200
Diabetes	1.46 (0.76–2.78)	0.253	—	—
Hyperlipidemia	0.69 (0.32–1.47)	0.336	—	—
Traumatic diseases	0.79 (0.37–1.69)	0.546	—	—
Malignant tumor	0.93 (0.66–1.32)	0.688	—	—
Biochemical patterns of DILI				
Hepatocellular type	0.98 (0.65–1.49)	0.940	0.94 (0.61–1.45)	0.772
Mixed type	1.11 (0.67–1.84)	0.689	1.11 (0.64–1.95)	0.708
Cholestatic type	Reference			
Accompanying symptoms				
Jaundice	0.80 (0.49–1.29)	0.360	—	—
Anorexia	1.23 (0.74–2.05)	0.425	—	—
Nausea	1.62 (0.95–2.79)	0.079	1.93 (0.98–3.81)	0.057
Vomiting	1.51 (0.70–3.23)	0.292	—	—
Abdominal discomfort	2.40 (1.32–4.37)	0.004	1.72 (0.84–3.51)	0.140
Rash	0.85 (0.37–1.93)	0.703	—	—
Severity grading				
Mild	Reference			
Moderate	1.19 (0.83–1.72)	0.347	0.86 (0.53–1.40)	0.534
Severe	0.50 (0.16–1.58)	0.237	0.64 (0.19–2.15)	0.467

a Community-acquired DILI was defined as a liver injury occurring in a community setting with the patient admitted to the hospital on the first liver biochemical test above the threshold.

b Liver malignancies were not included in underlying liver disease but were classified as malignant tumors.

Abbreviations: aHR, adjusted hazard ratio; CI, confidence interval; HR, hazard ratio; DILI, drug-induced liver injury; and PAC, pharmacist active consultation.

We compared the quality of DILI management between the PAC and no PAC patients in the PSM cohort (Table 5). All of the patients in the PAC group (reference) were considered to have received professional advice and be aware of the possibility of DILI. In contrast, not all patients in the no PAC group were aware of the possibility of DILI (100% vs. 70.7%, *p* < 0.001), and these patients did not benefit from expert consultation (100% vs. 36.7%, *p* < 0.001). PAC was associated with a higher rate of identification of suspect drugs (90.2% vs. 59.8%, *p* < 0.001) and timely withdrawal of medication (89% vs. 57.3%, *p* < 0.001). However, there were no significant differences between the two groups in consultation interval, liver function monitoring interval, number of hepatoprotective drugs used, and glucocorticoid use.

**TABLE 5:** Quality of medical care in the propensity score-matched cohort of drug-induced liver injury patients.

Management quality	No PAC service (*N* = 82)	PAC service (*N* = 82)	*p*-value
Expert consultation^a^	26 (31.7)	82 (100.0)^b^	<0.001***
Recognition of the possibility of DILI	58 (70.7)	82 (100.0)^b^	<0.001***
Identification of suspect drug	49 (59.8)	74 (90.2)	<0.001***
Timely discontinuation of the suspect drug	47 (57.3)	73 (89.0)	<0.001***
Expert consultation interval, h [IQR]^c^	27.4 [5.3, 78.6]	13.5 [6.4, 30.5]	0.294
Liver function monitoring interval, h [IQR]	72.9 [48.2, 116.1]	90.5 [69.2, 119.8]	0.239
Number of hepatoprotective drug use			0.337
0	10 (12.2)	7 (8.5)	
1	17 (20.7)	27 (32.9)	
2	31 (37.8)	32 (39.0)	
3	20 (24.4)	14 (17.1)	
4	4 (4.9)	2 (2.4)	
Glucocorticoid use	16 (19.5)	25 (30.5)	0.149

Data are presented as no. of patients (%) unless otherwise specified.

a Expert was defined as a pharmacologist or hepatologist.

b With the PAC group as a reference, all patients in the PAC group were considered as having received professional consultation advice and being aware of the possibility of DILI.

c In the no PAC group, only the matched 26 patients who received hepatologist consultation were assessed.

Abbreviations: PAC, pharmacist active consultation; IQR, interquartile range; and DILI, drug-induced liver injury.

## Discussion

We examined the utility of a PAC service provided by clinical pharmacists for the identification and management of patients with DILI. We found that proactive pharmacy consultation improved patients’ 180-day cumulative recovery rate. Adjusted Cox multivariate analysis, IPTW weighting, and PS matching further supported these results.

The cumulative rate of recovery over 180 days of follow-up was 86% in the no PAC group and 96% in the PAC group. Patients without the PAC service followed the natural course of recovery from DILI. The estimated probability of recovery by six months was previously reported as ranging from 0.46 to 0.93 in DILI patients with different clinical characteristics (Ashby et al., 2021), which is similar to the recovery rate observed in the no PAC group. PAC was associated with an approximately 10% increase in recovery rate. There are two possible explanations for this result. First, PAC improved the quality of medical care for DILI patients, which accelerated their recovery. Second, as this was a real-world study with possible confounders and selection bias, there may have been an imbalance in patient baseline and clinical characteristics between the two groups. In fact, some characteristics were imbalanced between the two original cohorts (Table 1). To minimize the impact of confounding factors on outcomes, we performed an adjusted Cox multivariate analysis and used IPTW and PS matching. The higher recovery rate of the PAC group remained robust after controlling for confounders, suggesting that it was mainly due to improved management of DILI.

In the PSM cohort, the coverage of specialist consultation in the no PAC group was just 30%, implying that most patients did not experience the benefit of evidence-based treatment guidelines. This could in theory be resolved if all patients with suspected DILI sought expert consultation with a hepatologist. However, as skilled doctors at tertiary hospitals in China are greatly overworked (Hu and Zhang, 2015), only a limited number of patients can receive such consultation. The clinical pharmacist, who has medication management skills, is uniquely trained to assist individual patients through effective dispensing of medications, which can prevent adverse drug-related outcomes (Mansur, 2016). Thus, clinical pharmacists can share the workload of skilled doctors by assuming the responsibility of expert consultation.

Based on the PSM cohort, we found that the PAC was associated with higher rates of DILI diagnosis and identification of suspect drugs, as well as timely drug discontinuation, which is the preferred management strategy for suspected DILI although it is predicated on correct identification of the causative drug (Yu et al., 2017; European Association for the Study of the Liver. Electronic address et al., 2019; Chalasani et al., 2021; Devarbhavi et al., 2021). To this end, and in order to provide appropriate recommendations, clinical pharmacists referred to the available evidence-based resources for the diagnosis and management of DILI (Isaacson and Babich, 2020). In some cases, an appropriate recommendation is not limited to accurate identification of the causative drug; a more challenging decision is that of drug continuation or rechallenge under the precondition of DILI. Drug rechallenge may be appropriate under the following circumstances: 1) when no safer alternatives are available, 2) the objective benefits exceed the risks, and 3) patients are fully informed and provide consent, adhere to their treatment for the duration of follow-up, and alert healthcare providers to symptoms of hepatitis (Hunt et al., 2017). Of the 108 patients in the PAC group, cautious drug rechallenge was recommended by the clinical pharmacist in 10 cases. These decisions were evidence-based and in accordance with guideline recommendations for specific drugs (e.g., hepatotoxicity related to immune checkpoint inhibitors or antituberculosis drugs) (Senousy et al., 2010; Remash et al., 2021).

In this study, clinical pharmacists were involved in the treatment of all patients in the PAC group, including the screening of alternative etiologies for DILI, attributing causality to a specific agent, deciding to continue or discontinue the drug, and administering appropriate drugs for DILI therapy. This is in line with the pharmacist’s responsibility to engage in comprehensive drug management and share the workload of clinicians. However, these are secondary to providing high-quality medical care to patients through PAC services. The present study also summarized DILI prevention and treatment strategies used at our institution that allows clinical pharmacists to correctly identify patients requiring attention and appropriate drugs in order to provide optimal pharmaceutical care.

To our knowledge, this is the first study to assess the impact of PAC on DILI patient recovery, and it had several advantages. Confounding factors were well controlled and the impact of PAC on 180-day recovery was demonstrated. Our results also showed a new way to manage DILI through clinical pharmacist involvement and can serve as a reference to medical institutions for improving the quality of medical care. However, this study also had several limitations. First, it was based on data from a retrospective review of medical records, and causality between the intervention (PAC) and the outcomes of DILI patients was assessed using the updated RUCAM, which is best applied to a prospective study design; thus, we could not ensure data completeness and high RUCAM scores. Second, because of the retrospective nature of the study, there may have been unrecognized confounding variables linking PAC and patient recovery. Third, this was a single-center study and the findings may not apply to other centers. Fourth, because our cohorts included many patients with other alternative causes of DILI categorized as “possible” in the RUCAM causality assessment, the identification of DILI patients is still ambiguous and the results remain controversial. Finally, the PAC service mainly targeted patients with mild to moderate DILI, while those with serious DILI were treated at the Hepatology Department by hepatologists and were not included in the analysis. Therefore, the effect of PAC on the recovery of patients with severe DILI is unclear and requires further validation in a well-designed study.

## Conclusion

Our study provides evidence that DILI patients can benefit from PAC services. Clinical pharmacists can share the responsibility of drug management for DILI with doctors by providing evidence-based treatment recommendations. Our findings can encourage greater pharmacist involvement in patient care and collaboration with other healthcare providers to improve the outcome for patients with DILI. We also recommend the use of the updated RUCAM in future DILI cases and similar studies to assist DILI patient identification and enrollment.

## Data Availability

The original contributions presented in the study are included in the article/Supplementary Material; further inquiries can be directed to the corresponding authors.
