# Supplementary material for: Impact of pharmacist active consultation on clinical outcomes and quality of medical care in drug-induced liver injury inpatients in general hospital wards: A retrospective cohort study
Source: Front Pharmacol. 2022 Aug 30;13:972800. doi: 10.3389/fphar.2022.972800 (PMC9468675; doi:10.3389/fphar.2022.972800)
Supplement: Supplementary file 1 [file DataSheet1.docx]

**Supplementary Table S1.** Drug-induced liver injury severity grading scale

| **Severity grading** | **Definition** |
| --- | --- |
| Mild | ALT ≥5 ULN or ALP ≥2 ULN and TBL <2 ULN |
| Moderate | ALT ≥5 ULN or ALP ≥2 ULN and TBL ≥2 ULN, or symptomatic hepatitis |
| Severe | ALT ≥5 ULN or ALP ≥2 ULN and TBL ≥2 ULN, or symptomatic hepatitis and at least one of the following criteria:  - INR ≥1.5  - Ascites and/or encephalopathy, disease duration <26 weeks, and absence of underlying cirrhosis  - Other organ failure considered to be due to DILI |
| Fatal/transplantation | Death or liver transplantation due to DILI |

Abbreviations: ALP, alkaline phosphatase; ALT, alanine aminotransferase; DILI, drug-induced liver injury; INR, international normalized ratio; TBL, total serum bilirubin; ULN, upper limit of normal.

**Supplementary Table S2.** Patients with other possible causes of liver injury

| **Group** | **Sex/age, years** | **Treatment** | **Type** | **Severity** | **Alternative causes** | **RUCAM** |
| --- | --- | --- | --- | --- | --- | --- |
| No PAC | M/50 | Valproate | Hepatocellular | Mild | Recent hemodynamic abnormality | 4 |
| No PAC | M/54 | Sevoflurane | Hepatocellular | Mild | Chronic hepatitis B | 3 |
| No PAC | M/77 | TCM | Hepatocellular | Moderate | Chronic hepatitis B | 5 |
| No PAC | M/74 | TCM | Hepatocellular | Moderate | Hepatitis E | 4 |
| No PAC | M/72 | TCM | Hepatocellular | Mild | Chronic hepatitis B | 3 |
| No PAC | M/25 | Valproate | Mixed | Mild | Recent hemodynamic abnormality | 3 |
| No PAC | M/64 | Apatinib | Cholestatic | Moderate | Chronic hepatitis B | 3 |
| No PAC | F/50 | Valproate  Methylprednisolone (HD) | Hepatocellular | Mild | Recent hemodynamic abnormality | 3 |
| No PAC | M/69 | Atorvastatin | Hepatocellular | Moderate | Chronic heart failure | 4 |
| No PAC | F/88 | TCM | Hepatocellular | Moderate | Cholecystitis | 5 |
| No PAC | F/53 | TCM | Hepatocellular | Moderate | Cholecystitis | 4 |
| PAC | M/76 | Valproate | Hepatocellular | Mild | Recent hemodynamic abnormality | 4 |
| PAC | F/39 | *Tongguanteng* injection | Hepatocellular | Mild | Liver malignancy | 4 |
| PAC | M/41 | Mosapride | Mixed | Mild | Recent hemodynamic abnormality | 3 |
| PAC | F/73 | Valproate | Hepatocellular | Moderate | Recent hemodynamic abnormality | 3 |
| PAC | M/64 | TCM | Hepatocellular | Mild | Chronic hepatitis B | 4 |
| PAC | F/55 | Total nutrient admixture | Cholestatic | Mild | Liver malignancy | 3 |
| PAC | M/63 | Fluconazole  Methylprednisolone (HD)  Levofloxacin | Mixed | Moderate | AOSD | 5 |
| PAC | F/51 | TCM | Hepatocellular | Mild | Liver malignancy | 4 |
| PAC | M/67 | Atorvastatin  Niacin | Hepatocellular | Mild | Heavy drinking | 4 |
| PAC | M/59 | Sivelestat | Hepatocellular | Mild | Recent hemodynamic abnormality | 4 |

Abbreviations: AOSD, adult onset still disease; F, female; HD, high-dose; M, male; PAC, pharmacist active consultation; RUCAM, Roussel Uclaf Causality Assessment Method; TCM, traditional Chinese medicines.

**Supplementary Table S3.** Causative drugs of drug-induced liver injury

| **ATC classification^†^** | **All patients**  **(N=260)** | **No PAC service**  **(N=134)** | **PAC service**  **(N=126)** |
| --- | --- | --- | --- |
| Antineoplastic and immunomodulating agents | 78 (30.0) | 37 (27.6) | 41 (32.5) |
| TCM | 52 (20.0) | 34 (25.4) | 18 (14.3) |
| Anti-infectives for systemic use | 40 (15.4) | 22 (16.4) | 18 (14.3) |
| Nervous system | 37 (14.2) | 21 (15.7) | 16 (12.7) |
| Cardiovascular system | 19 (7.3) | 5 (3.7) | 14 (11.1) |
| Systemic hormonal preparation, excluding sex hormones and insulin | 10 (3.8) | 7 (5.2) | 3 (2.4) |
| Musculoskeletal system | 10 (3.8) | 4 (3.0) | 6 (4.8) |
| Alimentary tract and metabolism | 8 (3.1) | 3 (2.2) | 5 (4.0) |
| Blood and blood-forming organs | 1 (0.4) | 0 (0.0) | 1 (0.8) |
| Other | 5 (1.9) | 1 (0.7) | 4 (3.2) |

^†^Anatomic or pharmacologic groups (first level) in ATC classification system of World Health Organization.

Abbreviations: ATC, anatomic therapeutic chemical; PAC, pharmacist active consultation; TCM, traditional Chinese medicines.

**Supplementary Table S4.** List of drugs prolonging recovery from drug-induced liver injury

| **Group** | **Sex/age, years** | **Drug** | **Type** | **Severity** |
| --- | --- | --- | --- | --- |
| No PAC | M/53 | TCM | Hepatocellular | Severe |
| No PAC | M/43 | Methylprednisolone (HD) | Hepatocellular | Mild |
| No PAC | F/39 | Azithromycin | Hepatocellular | Mild |
| No PAC | F/50 | Valproate | Hepatocellular | Moderate |
| No PAC | M/33 | Acetaminophen | Hepatocellular | Mild |
| No PAC | F/78 | Voriconazole | Cholestatic | Mild |
| No PAC | M/60 | Sulbactam, cefoperazone | Cholestatic | Mild |
| No PAC | M/67 | Atorvastatin | Hepatocellular | Mild |
| No PAC | M/45 | Vancomycin | Hepatocellular | Mild |
| No PAC | M/62 | Pembrolizumab | Mixed | Moderate |
| No PAC | M/65 | Pembrolizumab, paclitaxel, lobaplatin | Cholestatic | Mild |
| No PAC | M/61 | Sevoflurane | Hepatocellular | Mild |
| No PAC | M/62 | Methylprednisolone (HD) | Hepatocellular | Mild |
| No PAC | M/70 | Tigecycline | Cholestatic | Moderate |
| No PAC | M/25 | Valproate | Mixed | Mild |
| No PAC | F/39 | Sulbactam and cefoperazone | Hepatocellular | Mild |
| PAC | M/72 | Voriconazole, sulbactam, cefoperazone | Cholestatic | Mild |
| PAC | M/78 | Paclitaxel, sintilimab | Hepatocellular | Moderate |
| PAC | F/51 | Sintilimab | Hepatocellular | Mild |

Abbreviations: F, female; HD, high dose; M, male; PAC, pharmacist active consultation; TCM, traditional Chinese medicines.
